# Addressing blood-brain-tumor-barrier heterogeneity in pediatric brain tumors with innovative preclinical models

**DOI:** 10.3389/fonc.2023.1101522

**Published:** 2023-01-26

**Authors:** Elysse K. Morris, Sheena Daignault-Mill, Samantha J. Stehbens, Laura A. Genovesi, Anne K. Lagendijk

**Affiliations:** ^1^ Institute for Molecular Bioscience, The University of Queensland, St. Lucia, QLD, Australia; ^2^ The University of Queensland Frazer Institute, Faculty of Medicine, The University of Queensland, Brisbane, QLD, Australia; ^3^ School of Biomedical Sciences, University of Queensland, St. Lucia, QLD, Australia

**Keywords:** medulloblastoma, pediatric brain tumor, blood brain barrier, neurovascular unit, zebrafish, endothelial cells, microfluidics

## Abstract

Brain tumors represent the leading cause of disease-related mortality and morbidity in children, with effective treatments urgently required. One factor limiting the effectiveness of systemic therapy is the blood-brain-barrier (BBB), which limits the brain penetration of many anticancer drugs. BBB integrity is often compromised in tumors, referred to as the blood-brain-tumor-barrier (BBTB), and the impact of a compromised BBTB on the therapeutic sensitivity of brain tumors has been clearly shown for a few selected agents. However, the heterogeneity of barrier alteration observed within a single tumor and across distinct pediatric tumor types represents an additional challenge. Herein, we discuss what is known regarding the heterogeneity of tumor-associated vasculature in pediatric brain tumors. We discuss innovative and complementary preclinical model systems that will facilitate real-time functional analyses of BBTB for all pediatric brain tumor types. We believe a broader use of these preclinical models will enable us to develop a greater understanding of the processes underlying tumor-associated vasculature formation and ultimately more efficacious treatment options.

## Introduction

**Figure 1:**
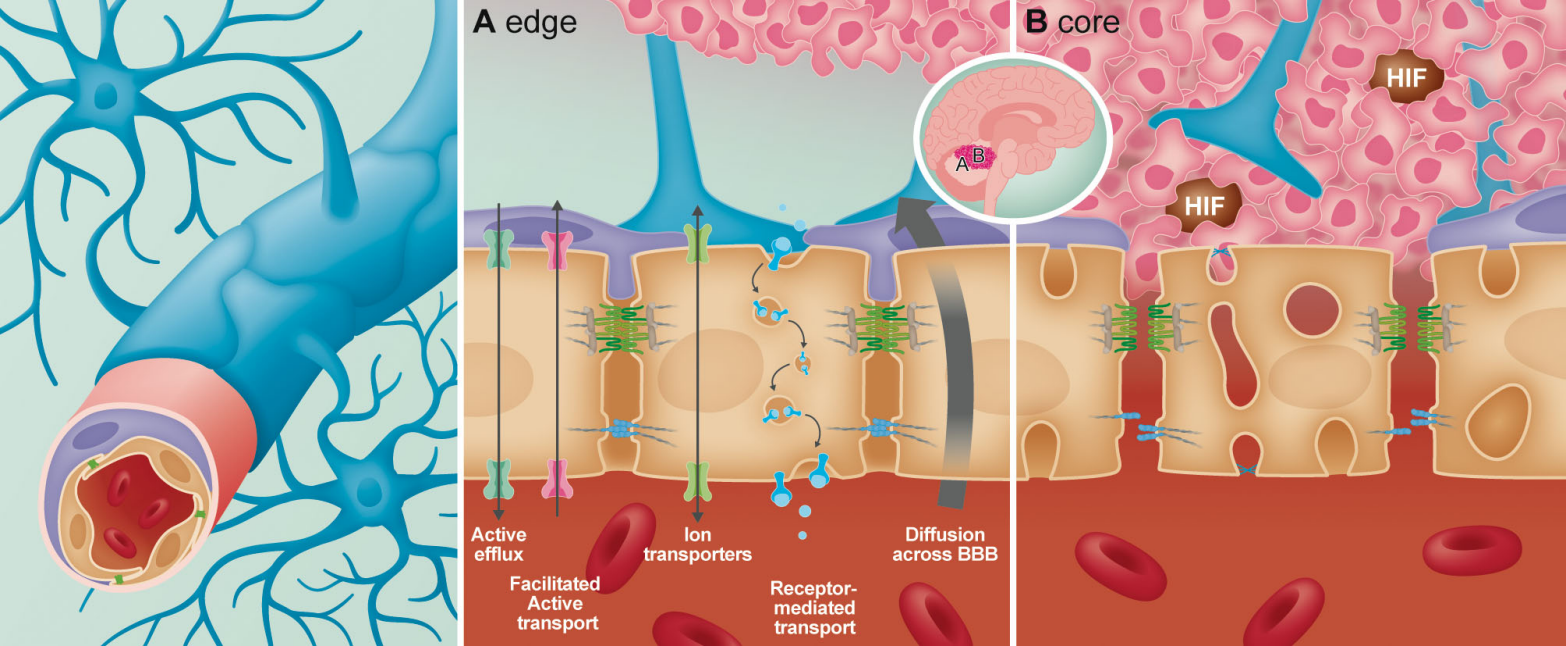
Overview of the structural and anatomical position of the cellular components forming the neurovascular unit (NVU) of the blood-brain-barrier (BBB). In the BBB the endothelial cells act as the interface between the circulating blood and the brain parenchyma. Interlocked with the endothelial cells *via* “peg-and-socket” connections are the pericytes which aid in maintaining and promoting BBB integrity. Additional components of the NVU are astrocytic endfeet that encapsulate all CNS capillaries for maintenance of the BBB. In the presence of a tumor, heterogeneity of the BBB from the edge **(A)** to the core **(B)** has been identified. **(A)** At the edge of the tumor, the BBB remains intact, with unperturbed transport across the endothelial cells (paracellular and carrier/receptor mediated transcytosis). At the core of the tumor **(B)**, permeability of the BBB increases due to loss of astrocytes and pericyte coverage. In the endothelial cells, junctional integrity diminishes *via* loss of tight and adherens junctional proteins, while endothelial fenestrations increase leading to enhanced permeability.

The BBB is established through physical and functional interactions of different cell types, referred to as the neurovascular unit (NVU) (1–3), including non-fenestrated endothelial cells (ECs), pericytes, astrocytes and microglia (2–4). In addition to these cellular components, the BBB is further supported by a specialized extracellular matrix (ECM) (5). Here we focus on the structure and function of these BBB components, highlighting complexities within tumor vasculature of pediatric brain cancer and advances in innovative vasculature modelling. For detail into physiological structure and function of NVU components we refer to **Table 1**. In the context of brain tumors, the BBB is commonly referred to as the blood-brain tumor barrier (BBTB) (3) and is generally thought to be more permeable and ‘leaky’ (3, 35) than the BBB under normal physiological conditions. Leakiness is generally considered to be a consequence of cancer cells disrupting the function or distribution of cells that make up the NVU (36, 37). This relationship is displayed in **Figure 1**. Clinically, BBB dysfunction in brain tumors is assessed by contrast enhanced magnetic resonance imaging (CE-MRI) using a gadolinium-based contrast agent (38). Studies over the past decade have highlighted that not all pediatric brain tumors alter BBTB similarly. Instead, BBTB function is heterogenous between tumor types as well as within individual tumors (39–42). Understanding BBTB heterogeneity will allow the development of more targeted and effective treatments of distinct tumors. Here we discuss observations made in the most common and also most lethal malignant pediatric brain tumors, Medulloblastoma (MB) and Diffuse Intrinsic Pontine Glioma (DIPG).

**Table 1:** Structure and function of neurovascular unit components.

	Structure	Function
**Endothelial Cells**	• Continuous monolayer of ECs that are tightly connected *via* transmembrane tight junction proteins (3, 6, 7) • ↑ Mfsda2 expression, ↓ transcytosis (8–10) • Lack desmosomes and fenestrae (6, 10)	• Facilitates bi-directional transport of substances between brain parenchyma and blood (9, 10) • Transport mediated *via* (6, 10–12): ○ Paracellular diffusion ○ Carrier and receptor mediated transcytosis (9, 11) • Secrete PDGF-B to recruit pericyte anchorage (10, 13)
**Astrocytes**	• Endfeet processes encapsulate all CNS capillaries and arterioles (3, 10) • Fine processes extend to synapses, nerve cell bodies, and nodes of Ranvier (7, 14, 15) • Astrocyte to astrocyte connection and communication *via* gap junctions (5, 14) • Two types (14–16): ○ protoplasmic astrocytes: uniform distribution within grey matter • Complex cells that envelope synapses and microvasculature ○ Fibrous astrocytes: distributed along white matter tracts • Contact nodes of Ranvier and Oligodendroglia	• Facilitate bi-directional signalling between ECs and neurons controlling blood flow and neural activity (3, 14, 17) • Regulator of ion and water homeostasis (3, 10, 14, 15) • Phagocytic functions: clearing synaptic debris and protein aggregates (15) • Promotes and maintains BBB integrity • Regulator of immune cell entry in the brain (17–19): ○ physiological (restrict) ○ pathological (promote)
**Pericytes**	• Envelop capillaries, highly abundant in the CNS (7, 10) • Connect with ECs *via* tight, gap and adherens junctions at peg-socket contacts (20, 21) • Physiologically static, can remodel upon loss of neighbouring pericytes (7)	• Promote and maintain angiogenesis *via* crosstalk with ECs (10, 21, 22) • Regulates expression of tight and adherens junction proteins in ECs, thereby controlling BBB permeability (22) • Regulate capillary blood flow *via* neuronal coupling (23, 24) • Redirect and modulate polarisation of astrocyte endfeet on capillary wall (13, 21)
**Microglia**	• Resident immune cells of the CNS (25, 26) • Symmetrical extension and retraction of processes allows for surveying of the environment during physiological conditions (27, 28) • Little turnover during physiological conditions, remain quiescent (18, 26) • Activation of resting microglia gives rise to M1 (pro-inflammatory) and M2 (anti-inflammatory) microglial (18)	• Survey and respond to pathophysiological stressors within the brain microenvironment to maintain homeostasis (18, 25, 28) • Mediate tissue repair, activation of inflammation, and neuronal degradation/repair (18, 28) • Rapidly respond to threats in pathological conditions, indicative of morphological changes and chemokine release (28, 29) • Secrete cytokines to upregulate EC adhesion molecules and activate an immune response (25)
**Basement Membrane**	• Highly organised protein sheet comprised of extracellular matrix proteins (5, 30–32) • Two types of BM in the brain: endothelial BM and parenchymal BM (5, 31, 33) • Highly dynamic structure (30, 31) • Two main families of ECM receptors that aid in cell-cell and cell-matrix connections: dystroglycans and integrins (5, 33)	• Provides structural support, cell anchoring and signalling transduction between cells (5, 30, 33) • Physical barrier restricting paracellular transport of cells and larger molecules and proteins (including infiltrating leukocytes) (5) • Mediates tissue shape and cell polarity (34)

## Recent findings highlighting the heterogeneity of the BBTB integrity in medulloblastoma

Medulloblastoma (MB) is the most common type of malignant pediatric embryonal tumor that forms in the cerebellum (43). Large scale genomics studies have defined MB into four major subgroups, namely Wingless (WNT), Sonic Hedgehog (SHH), Group 3 (Gp3) and Group 4 (Gp4). These subgroups have been further subdivided into a total of 13 subtypes with distinct molecular and clinical features (43–46). Standard treatment for children greater than three years of age includes surgery, radiation to the cranio-spinal axis and adjuvant chemotherapy (43, 44). WNT-driven MB displays the most favorable prognosis among the four subgroups (43, 44), in part attributed to their robust therapeutic response. CE-MRI studies clearly show variable BBTB integrity across MB subgroups. Solid enhancement indicative of increased BBTB permeability was observed in WNT MB. Heterogeneous contrast enhancement was observed in patients with SHH and Gp3 MB, indicating variable BBTB permeability among regions of the same tumor. Minimal or non-enhancing tumors with an entirely intact BBTB were characteristic of Gp4 (47–49).

Elegant preclinical studies are consistent with clinical observations, with heterogeneous BBTB permeability observed in various widely used preclinical MB mouse models. High-resolution dynamic CE-MRI analyses was recently used to evaluate the integrity and permeability of BBTB in a murine genetically engineered mouse model (GEMM) of SHH MB and patient-derived orthotopic xenograft (PDOX) models from SHH and Gp3 MB (39). BBTB integrity was highly variable in preclinical models of MB, with heterogeneous contrast enhancement observed in both SHH and Gp3 PDOX MB. The invasive front of SHH PDOX tumors displayed minimal contrast enhancement, indicating clear differences in vascular integrity where tumor tissue meets the brain parenchyma. A completely intact functional BBTB was observed in SHH MB tumors initiated in an independent GEMMs despite significant tumor burden (39, 50), consistent with findings from an additional GEMM model of SHH MB (50). Together, these findings indicate that distinct biological processes govern tumor vascularization in tumors that initiate endogenously (GEMM tumors) compared to ectopic tumor cell engraftment in an adult host mouse. Recently, lineage tracing studies performed in a GEMM of SHH MB showed that Sox2-positive MB cells extend protrusions to directly ensheathe nearby capillaries, similar to astrocytes endfeet, contributing to a more intact BBTB formation and function (51). These Sox2-positive MB cells that construct the BBTB were shown to be mechanoresponsive, with Piezo2-mediated signaling regulating both the state of Sox2-positive MB cells and the BBTB. Knockout of Piezo2 resulted in a compromised BBTB, as shown by intratumoural accumulation of systemically administered 1kDa Cadaverine and 70kDa Dextran (51), and extended survival in response to etoposide treatment compared to tumor-bearing controls. Another study systemically administered 70kDa tetramethylrhodamine (TMR)-dextran to demonstrate that a GEMM model of WNT MB lack a functional BBTB (50), consistent with the solid enhancement observed in patients with WNT MB. Increased BBTB permeability has been suggested to dictate the improved therapeutic response of WNT MB (52). Indeed, functional studies have shown that tumors with a permeable BBTB from a genetically engineered mouse model (GEMM) of WNT MB responded to vincristine, which does not penetrate well into normal brain tissue (52). Disruption of WNT signaling restored BBTB function, blocked delivery of vincristine to the tumor and rendering them resistant to therapy *in vivo*. Together these studies point to the influence of tumor genotype on the development of an intact BBTB and reinforce that *in vivo* preclinical models of MB faithfully recapitulate the intratumoral and intertumoral heterogeneity of the BBTB phenotype of primary MB.

Various alterations in a number of components of the NVU is likely contributing to BBTB dysfunction in MB. The degree of BBTB permeability observed by MRI was shown to be correlated to differences in the structural and subcellular features of tumor-associated vasculature (39). Intact tumor-associated vasculature of the GEMM model of SHH MB displayed organized, linear expression of junctional markers CD31 and CLDN5, outlining a continuous vessel structure with extensive astrocytic encircling (39, 51). Basement membrane components, pericyte coverage and tight junction proteins were significantly reduced in this model upon genetic deletion of Piezo2, leading to increased leakage of fluorescent dyes into the brain parenchyma (51). In SHH and Gp3 PDOX models, the BBTB is compromised with abnormal barrier features such as disorganized endothelial cell-cell junctions, minimal astrocyte coverage and the presence of fenestrated, immature endothelium as determined by the expression of fenestrae marker Plvap (53, 54), and the loss of Glucose transporter 1 (Glut1) (53). Similar defects were observed in tumor-associated vasculature of PDOX and GEMM WNT MB, with hemorrhagic, aberrant vascular networks displaying a non-BBB immunophenotype characterized by the ectopic expression of Plvap and the loss Glut1 (52). Transmission and scanning electron microscopy confirmed fenestrated pores connecting the luminal and abluminal compartments of endothelium of tumors from GEMM WNT MB, with disruption of endothelial tight junctions also confirmed (52). These vascular changes were not observed in tumor endothelium from GEMM SHH MB. Transcriptomic analysis also confirmed that this endothelium was more similar to peripheral endothelium with the down-regulation of endothelial tight junctional protein Cldn5 and Glut1, while endothelium from GEMM SHH MB was very similar to normal brain endothelium (52). Decreased pericyte coverage was also observed in tumors from GEMM WNT MB compared to normal coverage in GEMM SHH MB (52).

Whilst the above-mentioned studies have begun to explore functional differences in BBTB integrity and the structural and sub-cellular features of tumor-associated vasculature in MB, very little is known regarding the processes driving tumor vascularization. Several mechanisms of tumor vascularization have been defined including sprouting angiogenesis, intussusceptive angiogenesis, vessel co-option, vasculogenic mimicry and lymphangiogenesis (55). Histological analysis of brain tumor sections implies a role for angiogenesis and vascular mimicry in GEMM and PDOX tumor sections of SHH and Gp3 MB (39), with an earlier study identifying elevated Vascular endothelial growth factor (*VEGF)*, a principal angiogenic factor, in cell line xenograft Gp3 models and Gp3 MB patients (56). Given vasculature architecture has been shown to influence therapeutic response (52, 57), further characterization of tumor vasculature and the processes driving this in MB is necessary to ensure effective treatment of this disease.

## An intact BBTB represents a major hurdle in the treatment of Diffuse Midline Glioma

Diffuse Intrinsic Pontine Glioma (DIPG), more recently termed Diffuse Midline Glioma (DMG) (58), is a highly aggressive, lethal pediatric brain tumor that grows diffusely throughout the brainstem (59, 60). Surgical options are limited for DMG patients, largely due to the location of the tumors. Chemotherapy or other targeted therapies have not been shown to significantly improve survival rates for DMG patients (60, 61). One proposed explanation for the failure of systemically delivered therapies in DMG is due to the intact nature of the BBTB (59, 62, 63), as evidenced by the failure of contrast-enhancing agents to penetrate tumor tissue. Histological and molecular analyses of primary DMG, PDOX and *in utero* electroporation (IUE) mouse models, all revealed a minimal change in vascular phenotype within tumors compared to normal brain (58). The ECs displayed continuous expression junctional proteins CLDN5 and CD31, normal expression of the transporter Glut1 and did not express the pathological marker, Plvap. Extensive coverage by pericytes was also revealed and administration of 10kDa TMR-dextran in the IUE model, showing limited leakage. Together, these findings suggest that blood vessels are unaffected by the presence of DMG cells, possibly explaining why systemic therapy is not efficacious for this disease. Instead, novel delivery technologies such as small lipophilic drugs designed to cross an intact BBTB with minimal active efflux, are likely to be more successful. Another emerging technology is MRI-guided focused ultrasound (MRIg-FUS) that induces transient openings of the BBB by acoustic activation of circulating microbubbles. This technique has been shown to disrupt the BBB in a controllable manner in both animal models and in patients with high grade gliomas (64, 65). More recently, a similar approach was applied to target blood vessels in a PDOX model for DMG, revealing a significant increase in intra-tumoral doxorubicin concentrations and reduced tumor volume through MRIg-FUS (66).

The studies described here have begun to unravel the complexity of BBTB in MB and DMG and the relevance of modifying BBTB function for more effective, targeted treatment. However, before we can do this, a better understanding of the processes underlying tumor vascularization and the tumor-specific changes in NVU composition and BBTB function are urgently required. Next, we describe a range of innovative preclinical models that can be utilized to accelerate this understudied aspect of pediatric brain tumor biology.

## Innovative preclinical models to interrogate pediatric brain tumor vasculature

Preclinical mouse PDOX and GEMM models of MB and DMG are widely used as the gold standard for preclinical testing of novel therapeutics (67–69). Studies utilizing these models clearly show that a greater understanding of how tumor cells interact with each other, and their surrounding microenvironment is urgently needed. Further to this, we need to better understand how tumors orchestrate structural and functional changes in their associated vasculature before we can develop effective, targeted therapies for pediatric brain tumors. Such mouse models have further uncovered the important roles for astrocytes (70), pericytes (13, 71, 72) and microglial (73) cells for BBB development and integrity. However, the complex interplay of the NVU in the context of tumor progression and therapy response remains to be determined.

In murine models, non-invasive techniques such as positron emission tomography (PET), computed tomography (CT), and more commonly MRI, have uncovered changes in the BBTB (74). However, longitudinal dynamic high-resolution imaging is required to understand the interactions between the developing tumor and its associated microenvironment. Live imaging technologies have been developed for rodent brains (75, 76), however these approaches are incredibly costly and present with a number of technical challenges due to the location of MB and DMG within the cerebellum and the pons of the brain stem respectively.

## Dynamic modelling of tumor-vessel interactions in zebrafish

A vertebrate model that is rapidly developing as a robust model for cancer research is the zebrafish (77–81). A major reason for the expansion of zebrafish studies is the cost-effectiveness of the model due to the large number of offspring that can be obtained from a single mating and lower husbandry costs associated with zebrafish housing. Another great advantage is that zebrafish embryos and larvae are optically transparent and develop *ex utero*, allowing live visualization of organs and cells during zebrafish development. Although structural similarities of zebrafish and human proteins remain to be determined, whole genome sequencing has identified that approximately 80% of disease associated genes in humans are conserved in zebrafish (82). This finding combined with the ease of genetic manipulation in zebrafish (83–85), led to the development of genetically engineered zebrafish models for a range of cancer types (86). In addition to genetic models, xenografting approaches are also widely applied to study cancer biology in zebrafish. Since the adaptive immune system in zebrafish is fully functional from 28 days post fertilization (dpf) (87, 88), mouse or human cancer cells are tolerated without inflicting an immune response prior to this stage.

The brain is challenging to image in higher vertebrates due to the presence of a thick skull, zebrafish brains are much more accessible and thus researchers can visualize and monitor tumor cell behavior in space and time. The anatomy of zebrafish brains is comparable to that of mammalian brains, with significant homology in molecular signatures and structure of distinct brain regions (89). Notable differences however do exist in terms of size and organization of distinct brain regions (90–92). In terms of the BBB, live imaging of zebrafish vascular transgenic marker lines has been applied to establish that the BBB in zebrafish begins to form at three days post-fertilization (dpf) and is fully functional at 10 dpf (93, 94). The zebrafish NVU is made up of endothelial cells, pericytes and radial glial cells (95). The radial glial cells express key astrocytic markers including Gfap, glutamine synthase and Aqp4 (95) and therefore are considered to perform orthologous roles to astrocytes (95). Transgenic marker lines, labelling distinct cell types of the NVU have been developed (96–99), allowing live visualization of the morphology, abundance, and dynamic behavior of distinct NVU cell types simultaneously.

In the context of brain cancer, zebrafish provide a powerful model to monitor dynamic interactions between tumor cells and NVU cell types, therefore determining what pathological changes might be contributing to BBTB malfunction. To date a small set of genetic zebrafish models have been established to study pediatric brain tumors (100–102). One such model that recapitulates central nervous system primitive neuroectodermal tumors (CNS-PNETs) was developed by Solin and colleagues (102), whereby TALEN-mediated genome editing was applied to inactivate retinoblastoma1 (*rb1*). When placed on a *p53* null background, these *rb1* knockout fish developed malformations of the skull and lesions on the eye. Histological analysis of the lesions revealed that the majority resembled CNS-PNET tumors and others were glial-like (102). Others have tested whether oligoneural precursor cells (OPCs) could give rise to CNS-PNETs by overactivated NRAS/MAPK signaling exclusively in these *sox10* expressing cells (101). This resulted in the development of large lesions in 6-week old zebrafish with conserved genetic and histologic hallmarks. Since hundreds of zebrafish embryos can be derived from a single paired mating it is highly suited for drug screening, especially when screening water soluble compounds that can be added to the zebrafish water. The authors utilized a screening approach to show that addition of MEK inhibitors to the fish water could effectively inhibit the growth of CNS-PNETs upon orthotopic xenografting (101). Human derived pediatric brain tumor cells have not been studied using zebrafish xenografting, however, tumor cells derived from adult brain cancers have been grafted successfully showing that human cells can utilize the zebrafish brain microenvironment to proliferate and migrate from the initial injection site (87). One challenge to utilizing zebrafish for xenografting is the optimal physiological core temperature of 28°C. However, zebrafish can adapt to changes in temperature. Previous studies have successfully grown zebrafish at temperatures of up to 36°C for xenografting purposes without notable side effects (103–106). Whether changes in temperature might invoke more subtle changes in the biological response to xenografted cells remains to be determined (107, 108).

Nevertheless, we propose that with well-established methods in place to visualize NVU cell types in the zebrafish brain (96–99) and determine BBB permeability (95), the zebrafish model is perfectly positioned to enable in depth studies that will generate new knowledge into the fundamental aspects of pediatric tumor heterogeneity, drug responses, metastatic potential, and alteration of the microenvironment.

### Microfluidic tumor-vessel co-culture models

Traditional *in vitro* cell culture methods have been used in basic research for many years to study mechanisms of cancer cell growth and evaluate drug efficacy. Various pediatric brain cancer cell lines are currently commercially available, with the most widely published MB models such as D283MED, D341MED, D425MED, UW228-2 and DAOY propagated *in vitro* for decades. As seen with other widely utilised brain cancer cell lines (109), it is increasingly likely that the original molecular features and biological behaviour of tumor cells would have been lost, failing to recapitulate tumour heterogeneity. Additionally, it is well appreciated that these simplistic 2D cell culture systems do not model the spatial, cellular and chemical complexity of tumors and the associated TME (110), limiting the translational utility of these model systems.

3D spheroid models are being increasingly developed for a variety of pediatric brain cancers (111, 112) replicating elements of the tumor microenvironment such as a gradient distribution of nutrients, oxygen, pH, cell-cell and cell- extracellular (ECM) contact (113). 3D tumor spheres derived from a variety of pediatric brain cancers including PDOX models of Gp3 MB (114) and primary biopsy material from MB, Ependymoma, Glioblastoma and Astrocytoma patients (111) have been recently established. Whilst pediatric 3D tumor spheroid cultures represent an important advance for the field, they still lack several essential components of the brain specific TME such as the ECM and the diverse non-cancerous cells including endothelial cells (ECs), pericytes, fibroblasts, immune cells, astrocytes, neurons and microglia (115, 116). Advances in cellular engineering, biomaterials and biofabrication technologies have led to the development of co-culture platforms whereby 3D tumor spheroids can be grown in the context of blood vessels and biologically relevant ECM hydrogels (117). To model the brain endothelium specifically, distinct types of brain ECs have been employed to form so called 3D BBB models. Of particular interest are recent protocols that utilize induced pluripotent stem cell (iPSC) derived brain microvascular ECs (iBMECs) (118–122). This is because these iBMECs when grown with other NVU cell types have been shown to form a tight BBB with physiologically relevant barrier properties as measured by transendothelial electrical resistance (TEER) and extravasation of fluorescent dyes (118–122). The validation of structural and functional features of an intact BBB supports the utility of these models to monitor how tumor cells alter the BBB and how this impacts the efficacy of therapeutics. Further details on the overall benefits and drawbacks of BBB culture have been reviewed elsewhere (123–125).

Although 3D BBB-tumor co-culture models have not yet been implemented to study tumor spheroids derived from pediatric brain cancer, data from adult brain tumors supports the feasibility of this approach (126, 127). For glioblastoma, tumor spheres were grown alongside an iPSC-derived BBB to test combination therapies. Vincristine and doxorubicin, two anti-cancer drugs that do not cross the BBB, were added to the 3D vessel in combination with mannitol and gintonin, to temporally open the BBB (128). Drug uptake was significantly improved within the 3D glioblastoma spheroid in combinations with mannitol and gintonin addition which induces BBB permeability (128). With platforms and applications of organ-on-a-chip models now widely accepted for a variety of diseases (129–134), it is imperative that these models are adapted and transitioned for the study of pediatric brain cancer. These innovative *in vitro* platforms in conjunction with animal models will be highly important to better understand the molecular mechanism of such ailments and providing novel therapeutics that have been thoroughly tested to target tumors in the presence of a heterogeneous BBTB.

## Discussion

Ineffective drug delivery is thought to be a contributing factor underlying the failure of novel therapeutic strategies in early phase clinical studies after demonstrating significant preclinical anti-tumor efficacy. Understanding the plasticity of the BBTB by utilizing complementary pre-clinical models will help to overcome one of the biggest barriers to effective intratumoral drug penetration. This review has summarized recent discoveries that emphasize the relevance of BBTB heterogeneity in pediatric brain tumors. We propose that, in order to better understand how BBTB differences arise and what the functional consequences are for treatment, multi-disciplinary approaches that utilize innovative pre-clinical models hold great potential.

## Author contributions

EM, SD-M, SS, LG and AL conceptualized the idea of this review and co-wrote the manuscript. All authors contributed to the article and approved the submitted version.
